# Emodin-induced cell cycle arrest: A promising approach for cancer therapy (Review)

**DOI:** 10.3892/mi.2026.328

**Published:** 2026-06-19

**Authors:** Shreya Das, Jhansi Kompala, Kyle Laney, Sneha Pathak, Ravi Nayar, Sukant Khurana, Alfredo Ghezzi, Lakshminarayanan Karthik, Abhijit G. Banerjee

**Affiliations:** 1 IonCure Tech Pvt. Ltd., New Delhi 110085, India; 2 University of South Florida, Tampa, FL 33620, USA; 3 Sakra Premium Clinic, Bengaluru 560035, India; 4 Lyda Hill Institute for Human Resilience, University of Colorado, Colorado Springs, CO 80907, USA; 5 Department of Biology, UPR-Río Piedras, University of Puerto Rico, San Juan, Puerto Rico 00925-2535, USA; 6 Genomic Bio-Medicine Research and Incubation, Chhattisgarh (CGBMRI), Durg 491001, India

**Keywords:** emodin, cell cycle arrest, cyclins, tumor proliferation, checkpoint kinases, bioavailability, targeted therapy

## Abstract

Emodin is a naturally occurring anthraquinone being investigated for its anticancer potential due to its ability to modulate the cell cycle and inhibit tumor progression. The cell cycle is composed of a series of events that dictate cell growth and cell division. The G0/G1 checkpoint is the resting stage before the first gap phase (G1), whereas the G2/M checkpoint is the working stage from the second gap phase (G2) before mitosis begins. Emodin exerts its effects by the following means: Cyclins, which propel the cells through the cell cycle stages; cyclin-dependent kinases (CDKs), which are energy sources that, along with cyclins, push the cell cycle further; and finally, CDK inhibitors, such as p21 and p27, which can inhibit CDK activity. G0/G1 arrest is mediated by the suppression of cyclin D/CDK4 and cyclin E/CDK2 activity, whereas G2/M arrest results from the inhibition of cyclin B/CDK1 and the disruption of mitotic progression. G2/M arrest is further reinforced by the activation of the checkpoint kinases, Chk1 and Chk2. These kinases operate downstream of DNA damage sensors and effectors to maintain cell cycle blockade. The present review discusses the mechanisms of emodin-induced cell cycle arrest. In preclinical settings, emodin has been shown to be capable of suppressing tumor cell proliferation in multiple cancer models, both by itself and in combination with standard chemotherapy or radiotherapy. However, suboptimal bioavailability and metabolic instability are obstacles that have hindered its clinical application, with results being inconsistent among cancer types. Future studies are required to develop more efficacious drug delivery systems, identify predictive biomarkers and conduct strong clinical trials. Addressing these issues may position emodin as a viable cancer therapeutic option, either alone or in combination with current therapies.

## 1. Introduction

Cancer is an extremely complex disease characterized by the unregulated growth of cells, mostly due to deregulation of the cell cycle. The cell cycle is a tightly regulated and highly controlled process that depends on cyclins, cyclin-dependent kinases (CDKs) and the inhibitors, p21 and p27. For the cell to divide correctly, all regulatory molecules must function properly as any imbalance between these regulatory molecules may induce a state of tumorigenesis (1). The majority, if not all, cancers exhibit mutations or the overexpression of cyclins and CDKs, resulting in unrestricted proliferation. The entire cell cycle is a promising therapeutic target (2). By stalling cells at key checkpoint transition points in the cell cycle, such as at G0/G1 or G2/M, tumor cells can be prevented from completing mitosis, leading to growth inhibition and apoptosis (3). A number of natural compounds have been found to manipulate the cell cycle. One such effective chemical compound is emodin, which is an anthraquinone derived from several plants used in traditional medicine (4).

Emodin (1,3,8-trihydroxy-6-methylanthraquinone) has a planar aromatic structure that accounts for its interaction with a range of molecular targets. It is mainly extracted from medicinal plants, such as *Rheum palmatum* (rhubarb), *Polygonum cuspidatum* and *Aloe vera*, which have been traditionally used for centuries in Chinese and Indian Ayurvedic medicine (4). Traditionally, emodin-containing herbal products have been used as laxatives, anti-inflammatory agents, antimicrobials and liver protectants, suggesting its wide-ranging pharmacological potential beyond cancer therapy (5). Chemically, emodin has a low water solubility and moderate lipophilicity, which affect its bioavailability and metabolism *in vivo*, presenting a potential barrier to its therapeutic use (6). However, emodin has gained considerable interest for its multifunctional biological effects, which include antioxidant and pro-oxidant activities, the regulation of apoptosis, anti-angiogenic activity and the modulation of various signaling pathways, including PI3K/Akt, MAPK and NF-κB (6).

In terms of anticancer activity, emodin is effective, as it can regulate specific proteins involved in the cell cycle, inhibit threshold proliferation for tumors and induce apoptosis. Emodin increases CDK inhibitors (CDKIs), such as p21 and p27, that inhibit CDK activity and halt cell cycle progression altogether (7). Emodin also acts on a number of other signaling pathways which play a key role in the growth of cancer cells, rendering it a promising candidate for targeted cancer therapy (8). The present review discusses the mechanisms by which emodin induces cell cycle arrest, with a focus on its regulation of cyclins, CDKs and checkpoint pathways. The present review also addresses the effects of emodin on blocking the G0/G1 and G2/M phases of the cell cycle and its impact on tumor proliferation, thereby evaluating its efficacy as an anticancer agent and role in interrupting abnormal cell cycle progression.

## 2. Mechanisms of cell cycle regulation in cancer

The cell cycle is a process that regulates cell growth, DNA duplication, and ultimately, the division of cells into daughter cells. The cell cycle has five phases, which include G0 (resting phase), G1 (pre-DNA synthesis phase), S (DNA synthesis phase), G2 (pre-mitotic phase) and M (mitosis). The different cell cycle phases are tightly regulated by a network of regulatory proteins, but largely cyclins and CDKs. Cyclins are phase-specific activators that manage the passage of appropriate CDKs through the cell cycle by binding and activating CDKs. CDKIs help regulate the progression of the cell cycle, similar to that of a checkpoint, to limit uncontrolled division and errors of division (9).

In normal cells, controlled cell proliferation is maintained and regulated by the appropriate levels of cyclins, CDKs and CKIs. Nonetheless, the regulation of cyclin, CDK and CKIs in cancer is altered from normal levels due to genetic mutations and epigenetic changes. Genetic changes, including cyclins being overexpressed, the loss of CDKIs (p21 and p27) and mutations of tumor suppressor genes (*p*53 and *Rb*), cause the lack of regulated DNA duplication and cell division required for tumor growth (10). In addition to overactivation and genetic mutation, there is aberrant regulation at the transition from the G1 to the S phase of the cell cycle, and the G2 to M phase of the cell cycle, which can allow cancer cells to resist cell cycle arrest, accumulate DNA damage and proliferate in an uncontrolled manner (11).

Genomic instability driven by dysregulation of the cell cycle is a central hallmark of cancer that facilitates tumor evolution and therapeutic resistance. Uncontrolled cell division enables cancer cells to accumulate genetic alterations that promote survival, invasion and metastasis (12). In this context, targeting key regulators of the cell cycle, including cyclins, CDKs and checkpoint proteins has emerged as an effective therapeutic strategy. Natural products have gained attention as promising sources of such modulators, with compounds such as emodin demonstrating the ability to induce cell cycle arrest through multiple regulatory pathways (13). Understanding how emodin interacts with these critical regulators requires a detailed appreciation of the underlying molecular signaling mechanisms governing cell cycle progression.

The cell cycle is regulated at the molecular level by phosphorylation-dependent pathways. Cyclin-CDK complexes control the cell cycle by the phosphorylation of substrates, including the retinoblastoma (Rb) protein, resulting in E2F transcription factor release (14) and subsequent S-phase gene activation. In response to DNA damage and stress, tumor suppressors, including p53, lead to the induction of CDKIs (such as p21), resulting in cell cycle arrest (15). The disruption of these interconnected pathways underpins the uncontrolled growth of cancer cells and represents key targets for therapy. The following section discusses in detail how emodin influences these molecular mechanisms to induce cell cycle arrest in cancer cells.

## 3. Emodin-induced cell cycle arrest

**Figure 1:**
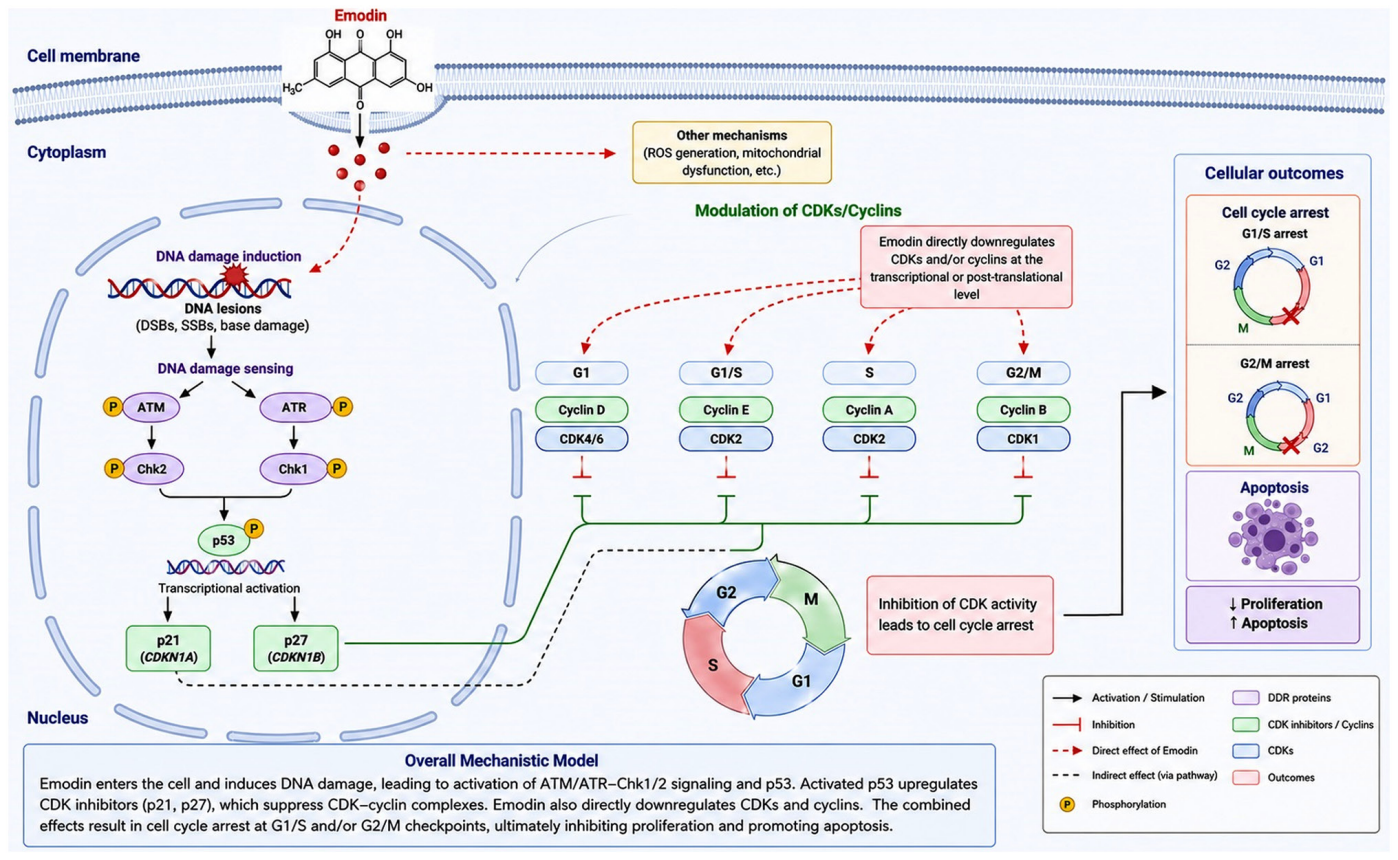
Mechanistic overview of emodin-induced DNA damage response and cell cycle regulation. Emodin enters the cell and induces DNA damage, activating the ATM/ATR-Chk1/Chk2 signaling cascade and p53. Activated p53 upregulates CDK inhibitors (p21 and p27), leading to the suppression of CDK-cyclin complexes. In parallel, emodin directly modulates CDKs and cyclins. These combined effects result in cell cycle arrest at G1/S and G2/M checkpoints and promote apoptotic outcomes.

Rather than exclusively modulating gene expression, emodin regulates phosphorylation-based regulatory networks, such as cyclin-CDK complexes, checkpoint kinases and tumor suppressors. By the combined effects of these mechanisms, emodin blocks key cell cycle transitions, such as the G1/S and G2/M checkpoints, to induce persistent cell cycle arrest and suppress tumor cell growth (Fig. 1) (16). Crucially, these actions are context-dependent and vary between cancer types due to their different genetic and signaling landscape.

### Primary molecular targets and upstream mechanisms of emodin

While the downstream consequences of emodin on cyclins, CDKs and checkpoint proteins are well documented, the primary targets of emodin remain under investigation. Available evidence indicates that emodin does not have a single main target, but rather displays multiple upstream pleiotropic effects (17). One of the most extensively studied mechanisms is the generation of cellular reactive oxygen species (ROS), which result in oxidative stress and DNA damage, activating the ATM/ATR pathway. This leads to the phosphorylation of downstream checkpoint kinases (Chk1 and 2) it's the stabilization of p53, resulting in cell cycle arrest (17,18). Beyond its effects on ROS-dependent signaling, emodin has been demonstrated to affect key growth and survival pathways, such as PI3K/Akt and MAPK, which affect upstream regulators of cyclin expression and cell cycle progression (19). Moreover, recent studies indicate that emodin may disrupt redox regulatory proteins such as MTH1, promoting increased oxidative DNA damage in cancer cells (20,21). The planar anthraquinone structure of emodin has also been shown to potentially interact with DNA or topoisomerase, potentially causing replication stress and checkpoint activation, although these interactions are less well understood (22). Taken together, these findings suggest that emodin functions as a multi-faceted agent that triggers upstream stress and signaling responses, which ultimately converge on the classical cell cycle regulatory pathways described in subsequent sections.

### Regulation of cyclins and CDKs

Cyclins are key proteins that progress the cell cycle through CDKs by forming complexes. It has been demonstrated that emodin alters the expression of several cyclins, leading to cell cycle arrest at checkpoints (23). Cyclin D and cyclin E facilitate the transition of cells from the G1 to the S phase, and emodin has been shown to downgrade their expression to limit DNA synthesis and help limit tumor cell proliferation (16). Additionally, the downregulation of cyclin A and cyclin B helps regulate the S and G2/M transition, which results in the additional blockade of the cell cycle, mitotic progression and limitation of tumor growth. CDKs, namely CDK2, CDK4 and CDK6 are key regulators of cyclin activity, and are often overexpressed in several forms of cancer (24). Emodin has been shown to inhibit the activity of these kinases, preventing them from interacting with their cyclins, thereby arresting progression through the cell cycle (25). Moreover, these kinases regulate the phosphorylation of the Rb protein, which is crucial for cell progression, keeping the cells from entering a proliferating state, thus forcing the cells into quiescence or arrest (26). The loss of CDK activity ultimately leads to growth suppression and the increased susceptibility of cancer cells to apoptosis.

CDKIs, such as p21 (Cip1/Waf1) and p27 (Kip1), function as tumor suppressors by negatively regulating CDK and enforcing cell cycle checkpoints. Emodin modulates the expression of those inhibitors to further inhibit CDKs 2 and 4, in addition to its direct impact on the kinases themselves (27). The elevated levels of CKIs further induce a cellular arrest in the G0/G1 phase of the cell cycle, preventing cells from entering into the S phase. Notably, p21 has also been demonstrated to inhibit CDK activity in a p53-independent manner, permitting emodin to maintain its effects against cancer cells even in tumors lacking functional p53 signaling (28). By simultaneously modulating the functions of cyclins, CDKs, and CKIs, emodin disrupts the cancer cell cycle, inhibits cancer cell proliferation and promotes dormancy (Table I) (29).

### G0/G1 phase arrest

The G0/G1 phase represents a bottleneck in the cell cycle where cells commit to initiating the replication process of their genome or enter a quiescent state. Cancer cells avoid proper regulation of the G1-S transition to undergo unwarranted and uncontrolled division processes (30). Emodin has been reported to induce G0/G1 arrest by disrupting regulatory proteins that control cell cycle checkpoints, which ultimately halts tumor growth and improves the therapeutic potential of anticancer treatments (31). The mechanism of action of emodin on G0/G1 arrest involves modulating cyclins, CDKs and tumor suppressor pathways. Key drug-mediated actions involve the inhibition of the cyclin D/CDK4 and cyclin E/CDK2 complexes, which help drive direct the progression of cells through the G1 checkpoint (32). With decreased reliance on CDK complexes, emodin diminishes the phosphorylation of the Rb proteins, permitting transcription factor E2F to release cells to enter S-phase. As a consequence, cells are left in the G0/G1 phase, indefinitely delaying DNA replication.

Cyclin D and CDK4/CDK6 are complexed to regulate the G1 phase transition, while cyclin E and CDK2 regulate the later G1 phase transition into S-phase (33). The relevant cyclins are typically overexpressed in a wide array of cancers, leading to high rates of proliferation (34). Emodin significantly reduces the expression levels of Cyclin D and Cyclin E as well as their respective CDKs (35). Since emodin down-regulates CDK activity, there is less phosphorylation of the Rb protein. This stalls the G1-S transition and can result in arrest of the cancer cell. Further characterization of emodin demonstrates that it increases the proteasomal degradation of cyclin D1, increasing the effectiveness of halting cell progression. Emodin arrests cells by blocking these relevant regulatory complexes, assuring tumor cells do not progress to the first checkpoint of the cell cycle and decreasing the ability of cancer cells to proliferate (36).

Protein p53 is a major tumor suppressor that regulates the G1 checkpoint, inducing the cell cycle in response to damaged DNA or other stressors. Emodin has been shown to induce p53 activation (37), resulting in a transcriptional upregulation of p21 (Cip1/Waf1) and p27 (Kip1) (38), both of which are strong CKIs. p21 and p27 have affinity to bind with CDK2 and CDK4 to prevent them from binding to cyclins, which inhibits cell cycle progression (39). Emodin-mediated p53 activation can further serve to upregulate p21 expression, reinforcing its effect on CDK inhibition and retaining G1 arrest. It can also promote p21 expression in a p53-independent manner, allowing treatments to be effective in p53-mutated cancers that are inherently resistant to traditional therapies (40). Emodin can enforce G0/G1 arrest through concurrent downregulation of cyclins/CDKs and upregulation of CKIs (Table I), leading to decreased proliferation of cancer cells and increased cancer cell sensitivity to apoptosis (41). Overall, these data suggest that emodin is a promising therapeutic agent in cancers linked to defects in cell cycle control.

### G2/M phase arrest

The G2/M checkpoint is a key regulatory checkpoint in the cell cycle to ensure that cells do not continue to mitosis with damaged or unreplicated DNA. Emodin has been shown to cause G2/M phase arrest in a number of cancer cells, effectively halting their proliferation and inducing prior steps along apoptotic pathways (42). G2/M arrest likely occurs due to the downregulation of the cyclin B/CDK1 complex, the activation of checkpoint kinases (Chk1/Chk2), and other steps that are interfering with mitotic progression (43). The cyclin B/CDK1 complex is critical in regulating entry into mitosis, and cancer cells frequently dysregulate this process, resulting in uncontrolled proliferation (44). Emodin reduces the expression of cyclin B1, and also inhibits the activity of CDK1, stopping cells from moving from the G2 phase into mitosis (45). Emodin has been shown to decrease the phosphorylation of CDK, required for its activation, and to contribute to G2 arrest for a prolonged duration (46).

Furthermore, in addition to inhibiting cyclin B/CDK1, emodin also interferes with mitotic spindle formation, leading to abnormal chromosome segregation (46,47). This results in a lengthened duration of mitotic arrest, which eventually leads to mitotic catastrophe and apoptotic cell death. Emodin induces DNA damage, activating the ATM/ATR signaling pathways, confirming G2 arrest (48). Given the accumulation of DNA damage and stalled replication forks, the cell is warned of the genetic instability and subsequently stops in mitosis, not allowing unstable and damaged cells to divide. Chk1 and Chk2 are two major checkpoint kinases that sense DNA damage and replication stress. Emodin has been reported to trigger the activation of Chk1/2, leading to the phosphorylation of Cdc25C, a key phosphatase in CDK1 activation (49). When Cdc25C is phosphorylated, it is retained in the cytosol, making it unable to induce mitotic entry. In addition to Cdc25C retention, Chk1/Chk2 ensures p53 stabilization, a key tumor suppressor, which leads to additional transcription of p21 and GADD45 to maintain G2/M arrest (50). Through these mechanisms, emodin is able to cease tumor cell proliferation and is a promising agent to impact anticancer therapies through cell cycle regulation (Table II).

Nonetheless, questions remain regarding potential toxicity to normal cells and the necessity for accurate dosing strategies to increase efficacy while decreasing side effects. Although emodin has demonstrated activity across multiple cancer models, its impact depends on cancer type, genetic mutations, and the tumor microenvironment. Emodin monotherapy has demonstrated partial potential in pancreatic cancer (51) and glioblastoma (52) (Table II), which may be due to inherent mechanisms of resistance, efflux of the drug, or other compensatory survival mechanisms. Although the efficacy of adding the chemotherapy agent, 5-fluorouracil, has been documented *in vitro* and *in vivo* (53-55), there is no uniformity of its effectiveness depending on cancer type, bioavailability issues and toxicity (56). Targeted delivery systems, combination therapy and mechanisms need to be incorporated in future research to maximize the capacity for emodin as a therapeutic in cancer.

## 4. Impact on tumor cell proliferation

The ability of emodin to modulate the cell cycle has implications for tumor suppression, mostly through inducing cell cycle arrest, blocking proliferation, and promoting both apoptosis and cell death pathways. In numerous studies (57-61), emodin has shown promise for anticancer potential. However, variations in response across tumor types and pharmacokinetic limitations highlight the need for additional studies (62).

### In vitro efficacy

**Figure 2:**
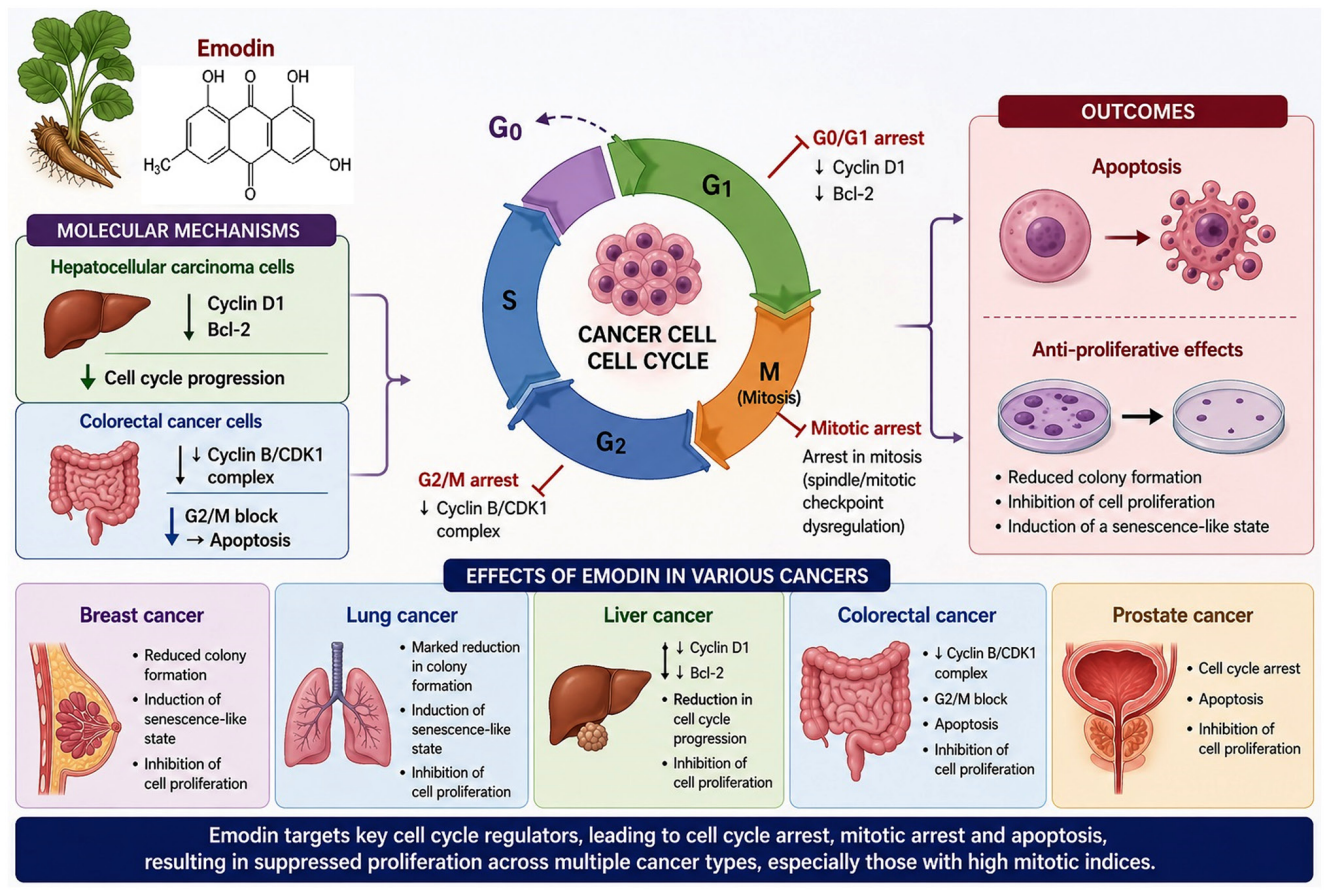
Emodin exerts potent anti-proliferative effects across multiple cancer types by disrupting key regulators of the cell cycle and survival pathways. It induces cell cycle arrest at G0/G1 and G2/M phases, suppresses mitotic progression, and promotes apoptosis through modulation of proteins, such as cyclin D1, Bcl-2, and the Cyclin B/CDK1 complex. These molecular effects are particularly evident in hepatocellular and colorectal cancer models, leading to impaired cell cycle progression and increased cell death. Additionally, emodin reduces colony formation and induces a senescence-like state in highly proliferative cancers, including breast, lung, liver and prostate cancers.

Emodin has been shown to inhibit cell proliferation in a number of *in vitro* cancer studies, including in breast, lung, liver, colorectal and prostate cancers (63-66). The emodin- mediated suppression of cell proliferation can result in G0/G1 or G2/M arrest, arrest in mitosis and can trigger apoptosis (Fig. 2).

In hepatocellular carcinoma cells, emodin has been reported to suppress Cyclin D1 and Bcl-2, causing a significant reduction in the cell cycle (67). Likewise, emodin also suppresses the migration and growth of the colorectal cancer cell lines (SW480 and SW620) by inhibiting the Wnt signaling pathway, and downregulating related genes and proteins, such as β-catenin, and Cyclin D1(68). The impact on cell cycle, and growth, is even more evident in cancers with very high mitotic indices, such as aggressive breast and lung cancers, which has shown a great reduction in colony formation and induction of a senescence-like state (69). However, not all cancers are equally sensitive to emodin. It has been found that cancer cells with functional p53 are more sensitive to emodin treatment, while cancer cells lacking p53 are relatively resistant to emodin-induced arrest (70). These results indicate that the effect of emodin is highly dependent on the genetic background of the tumor.

### In vivo tumor suppression

*In vivo* studies using tumor xenograft models have also demonstrated the anticancer effects of emodin (71,72). Emodin-treated tumors exhibit slower growth, increased apoptosis and lower proliferative indices. However, the effects are generally less pronounced than *in vitro*, due in part to poor pharmacokinetics, such as low bioavailability and rapid clearance (73,74). These results highlight the challenges of translating *in vitro* success to *in vivo* efficacy.

### Synergistic effects with chemotherapy and radiotherapy

Emodin has also been investigated as a chemosensitizer (chemotherapy) and a radiosensitizer (radiation) to improve the efficacy of conventional cancer therapies. It has demonstrated additive or synergistic effects with doxorubicin, cisplatin and paclitaxel, increasing cancer cell sensitivity to drug-induced cell death (75,76). This may be partly related to the ability of emodin to inhibit DNA repair and induce oxidative stress, rendering cancer cells more vulnerable to damage from chemotherapy. Emodin has also been shown to increase radiosensitivity by blocking DNA repair processes and increased ROS production (77). Pre-clinical studies have demonstrated an enhanced tumor suppression when emodin is used as an adjuvant to radiation therapy in lung and cervical cancer models (78,79). These results indicate that emodin could be used as an adjuvant therapy in cancer treatment.

## 5. Emodin and the regulation of non-coding RNAs

Recent investigations have indicated that emodin regulates non-coding RNAs, such as microRNAs (miRNAs/miRs) and long non-coding RNAs (lncRNAs), that have been shown to play a role in the cell cycle (80,81). For instance, miR-34a is a known tumor suppressor that upregulates p21, an inhibitor of CDKs, while miR-21 is an oncogenic miRNA whose targets include tumor suppressors such as PTEN (82,83). There is evidence to indicate that emodin can increase miR-34a and decrease miR-21 expression to further inhibit CDK activity and induce cell cycle arrest (84). In addition, oncogenic lncRNAs such as MALAT1, which is upregulated in aggressive cancers, promote cyclin expression and the cell cycle. Thus, based on the accumulating evidence of the targeting of the MALAT1 by small molecules (85), it would be interesting to assess the potential of emodin to modulate the stability and function of MALAT1 in cancer cells.

## 6. Epigenetics and cell cycle arrest

In addition to its epigenetic regulation of the cell cycle, emodin exhibits direct kinase inhibition. Histone acetylation and methylation play a pivotal role in modulating the expression of key cell cycle regulators, including the crucial CDKIs p16INK4A and p21. Emodin has demonstrated the ability to modulate histone deacetylases, resulting in increased histone acetylation and manipulation of tumor suppressor gene expression that mediates G1/S arrest (86,87). The re-activation of tumor suppressors and re-establishment of RB pathway control that prevent uncontrolled proliferation are influenced by DNA methylation. Emodin can regulate the reactivation, particularly at the p16INK4A locus (88). Emodin also blocks the activity of EZH2 chromatin remodeling enzymes, which silence tumor suppressor genes by trimethylating H3K27, further confirming its ability to induce cell cycle arrest (89,90).

## 7. Emodin in alternative cell cycle models

While it is known that emodin can lead to G1 and G2/M cell cycle arrest (91), further studies are required to investigate whether emodin can also induce other forms of cell cycle arrest, such as polyploidy and mitotic catastrophe. Certain natural products can induce endoreduplication, rendering cells polyploid and can ultimately trigger apoptosis or permanent cell cycle arrest (92). If emodin prevents mitotic spindle assembly, it may induce mitotic catastrophe (47), as in the case of rapidly dividing cancers, such as glioblastoma (93), and pancreatic cancer (94). Differentiating between cell quiescence and cell cycle arrest is important for therapeutic outcomes. The majority of chemotherapeutic agents induce G0 quiescence, which can be reversed and resumed upon drug removal. However, if emodin induces an irreversible senescent-like state, this would be a more sustained antitumor effect (95). Further research is required to establish whether the effects of emodin are reversible or induce irreversible cell cycle arrest (96).

## 8. Therapeutic implications

Emodin may be used as an adjuvant and single agent against cancer, particularly as it induces cell cycle arrest and high levels of sensitization to chemotherapy/radiation (78). Such multifaceted actions indicate that emodin functions as a pleiotropic bioactive agent, which can modulate various biological and pathological processes, thereby providing a broader perspective of its use in cancer treatment (25).

### Improving bioavailability

However, despite its potent anticancer properties, the therapeutic use of emodin is limited by its low water solubility, high metabolic rate and low systemic availability (97). To address these challenges, novel drug delivery strategies have been explored and tested in preclinical studies. Nanoparticles, such as polymeric nanoparticles and lipid-based nanoparticles have shown improved pharmacokinetics, selective tumor accumulation and higher intracellular drug concentrations (31). Liposomal formulations of emodin have also been reported to enhance its stability, decrease its systemic toxicity and enable controlled release of the drug in tumors (98,99). Moreover, nanoemulsions and solid lipid nanoparticles have been investigated to improve the solubility and permeability of emodin, leading to improved *in vivo* efficacy (100,101). These nanocarriers not only shield emodin from premature degradation, but also facilitate passive or active targeting of tumors through enhanced permeability and retention (EPR) mechanisms (102). These promising results in xenografts and other preclinical models have generated continuing research on these specific nanocarrier systems and quantitative therapeutic benefits (100,103).

Several pre-clinical studies have subsequently provided quantitative proof of the efficacy of nanocarrier-based emodin delivery systems (31). PEGylated liposomal emodin formulations, for instance, exhibit prolonged circulation time and improve tumor accumulation in xenograft mouse models than free emodin, leading to a significantly higher suppression of tumor growth with less systemic toxicity (104,105). Likewise, polymeric lipid nanoparticles loaded with emodin were found to enhance its oral bioavailability and cellular uptake and therefore, apoptotic activity was increased in hepatocellular carcinoma models (106). It has also been shown that polymeric nanoparticles based on poly(lactic-co-glycolic acid) possess sustained drug release property and enhanced intracellular retention resulting in increased antiproliferative activity of emodin in breast and hepatocellular carcinoma cells (107,108). In another method, nanoemulsion based formulation enhanced the aqueous solubility and permeability, resulting in higher *in vivo* antitumor activity and pharmacokinetic stability (109,110). Overall, these results suggest that the nanocarrier system can significantly counteract the inherent pharmacological limitations of emodin and enhance its translational potential in cancer therapy.

### Structural modifications and derivatives of emodin

The structural modification of emodin is another promising avenue with which to improve its therapeutic efficacy. Derivatives with modified structures, such as glycosylated compounds (e.g., emodin-8-O-β-D-glucoside) have shown enhanced solubility, stability and anticancer efficacy compared with the parent compound (7). Furthermore, the development of new emodin analogs seeks to improve pharmacokinetics, while retaining or boosting biological activity. Prodrug strategies have also been explored to enhance absorption and selectivity (110,111). Such modifications can increase cell membrane penetration, decrease enzymatic degradation, and increase plasma half-life. Overall, these types of structural modifications play a crucial role in addressing the drug development challenges of emodin to facilitate its clinical development.

### Clinical translation and current gaps

Although emodin has shown promising anticancer activity in preclinical studies, it has yet to be fully developed for clinical use. Currently, there are no reported clinical trials testing emodin as a single agent for cancer treatment. This underscores the need for effective translation of *in vitro* and *in vivo* results to clinical application. The lack of clinical evidence may be due to issues surrounding its bioavailability, pharmacokinetics and toxicity at higher doses (97). However, emodin is still being explored in combination treatments and as a scaffold for the development of more potent analogues. Future studies are required to focus on conducting proper preclinical studies with improved formulations, followed by well-conducted clinical trials to determine safety, efficacy and effective dose in humans. Overcoming these challenges is crucial to harnessing the full therapeutic potential of emodin in cancer treatment.

### Safety profile and comparison with conventional chemotherapy

While emodin has shown potential in cancer treatment, preclinical research has suggested that it may display dose-related toxicity, such as liver toxicity and gastrointestinal irritation, likely due to its pro-oxidant effect and metabolic instability (36). Furthermore, emodin has also been shown to trigger oxidative stress in normal cells at higher doses, which could limit its therapeutic index and lead to potential side-effects (112). Nevertheless, emodin has several advantages over classical chemotherapeutics, such as doxorubicin, cisplatin and paclitaxel. Conventional chemotherapy agents are frequently accompanied by severe side-effects, including myelosuppression, cardiotoxicity, neurotoxicity and multidrug resistance (80). However, emodin, being a natural product, displays a relatively selective cytotoxicity against cancer cells in a number of preclinical studies, owing to its ability to target cancer-specific vulnerabilities, such as elevated ROS levels and altered signaling pathways (8,49). Additionally, its ability to target multiple pathways also diminishes the potential for resistance development observed with single-target chemotherapies. While some studies and reviews have explored the potential for clinical use, these are largely speculative, and there is a clear gap between experimental findings and clinical development (6,25,78,80,100). Thus, comprehensive pharmacokinetic evaluations, toxicity assessments and early clinical studies are crucial to confirm the safety and efficacy of emodin-based treatments in humans.

## 9. Conclusion and future directions

Emodin has clocked into the potential of being a very effective natural agent that has an anticancer effect, particularly when causing cell cycle arrest and the contraction of tumors. Emodin blocks the uncontrolled growth of cancer cells by affecting important regulators of the G0/G1 and G2/M stages, including cyclins, CDK, p21, p27 and checkpoint kinases (Chk1/Chk2) (38,110). More recent studies have indicated that emodin also regulates non-coding RNAs, including miRNAs and lncRNAs, epigenetic modifications and alternative cell cycle pathways (81). These are all critical, yet currently unexplored pathways that may amplify its anticancer properties. The capacity of emodin to regulate miRNAs, such as miR-34a and miR-21, modify DNA methylation and histone acetylation, and cause polyploidy or mitotic catastrophe, render it a versatile cancer therapy method.

Some identifiable challenges have prevented this current bench research from being translated into clinical use, despite the potential. Emodin has limited bioavailability, is rapidly metabolized and has poor solubility. Nanoparticle delivery systems, structural modifications and newer formulations may help improve its limitations, and may lead to more effective therapies. Future studies are warranted to evaluate the safety and efficacy of emodin for humans through well-designed preclinical and clinical studies. Investigating its potential synergistic effect with chemotherapeutics, immunotherapy and/or targeted therapy also warrants further evaluation. By addressing present limitations and fully utilizing its numerous mechanisms of action (e.g., cell-cycle arrest, epigenetic effects and/or modulating non-coding RNAs), emodin may emerge as a critical component of modern anticancer therapeutics.

## Figures and Tables

**Table I:** Key cell cycle proteins modulated by emodin.

Protein	Effect	Functional consequence	Cancer model	Authors, year of publication (Refs.)
Cyclin D1	↓	Reduced G1 progression; proteasomal degradation ↑	Breast cancer (MCF-7); hepatocellular carcinoma	Sui *et al*, 2014(35); Subramaniam *et al*, 2013(67)
Cyclin E	↓	Inhibited S-phase entry; CDK2 partner lost	Colorectal cancer (HCT116); gastric cancer	Li *et al*, 2022(7); Liu *et al*, 2023(8)
CDK2	↓	Reduced kinase activity; Rb Ser811 not phosphorylated	Breast cancer (MCF-7); cervical cancer (HeLa)	Hsu and Chung 2012(28); Lande *et al*, 2023(45)
CDK4	↓	Impaired Rb Ser780/795 phosphorylation; G1 arrest	Hepatocellular carcinoma (HepG2); breast cancer	Subramaniam *et al*, 2013; (67); Sui *et al*, 2014(35)
CDK6	↓	Cyclin D1-CDK6 complex lost; G1 checkpoint enforced	Multiple cancer types	Vermeulen *et al*, 2003(26); Liu *et al*, 2023(8)
p21 (Cip1/Waf1)	↑	CDK2/CDK4 active-site blockade; p53-dependent and -independent	Cervical cancer (HeLa); lung cancer (NSCLC, A549)	Lande *et al*, 2023(45); Wahi *et al*, 2021(20)
Cyclin D1	↓	MCF-7 inactivation; MDA-MB-231 migration blocked	Breast cancer; colon cancer cells	Okon *et al*, 2023(69)
CDK2/Cyclin B1/*ERCC1*/AKT	↓	DNA damage; γH2AX foci; p53 stabilization	Multiple cancer types	Akkol *et al*, 2021(49)

↑, upregulated; ↓, downregulated; CDK, cyclin-dependent kinase. Concentrations are approximate effective/IC_50_ doses from cited studies.

**Table II:** Summary of emodin-induced cell cycle arrest across cancer models.

Cancer type	Cell line/model	Phase arrest	Key targets/pathways	Conc.^a^	Clinical relevance	Authors, year of publication (Refs.)
Hepatocellular carcinoma	HepaRG; HepG2	S and G2/M^b^	Cyclin A/CDK2 inhibition; MAPK/P13K/AKT	20-60 µM	Inhibited HepaRG proliferation; induced cell cycle arrest and apoptosis through the ROS- mediated mitochondrial pathway	Dong *et al*, 2018(77); Yin *et al*, 2022(83)
Colorectal cancer	HCT116; SW480	G1/S^c^	CDK1/CDK2 down- regulation; Rb inhibition	30-80 µM	Suppresses cell proliferation; inhibits tumor growth in xenograft model	Li *et al*, 2022(7)
Breast cancer	MCF-7 (ER^+^); MDA-MB-231 (TNBC)	G0/G1^b^	ERα^–M^APK/Akt-Cyclin D1/Bcl-2 pathway; p53- dependent arrest; CD155 downregulation	20-75 µM	TNBC subtypes sensitive; p53 status predictive of response	Sui *et al*, 2014(35); Fang *et al*, 2019(29)
Lung cancer	A549 (NSCLC)	G0/G1^b^	MTH1 inhibition; ROS augmentation; colony formation inhibition; senescence-like state induced	20-60 µM	Cisplatin sensitization; combination therapy potential; radiosensitization reported	Wahi *et al*, 2021(20); Zhang *et al*, 2022(78)
Pancreatic cancer	PANC-1; BxPC-3	G0/G1 (limited)^d^	Partial CDK4 suppression; p16/*RASSF1A* demethylation synergy with 5-Aza-CdR	40-100 µM	Resistance via KRAS pathway; requires delivery optimization	Pan *et al*, 2016(88); McDonald *et al*, 2022 *(*97)
Glioblastoma (GBM)	LN18/LN428, U87	G2/M (partial)^d^	Boosts neutron induced apoptosis; AhR signaling pathway	50-120 µM	Combined therapy promoted PARP1 fragmentation and caspase-3 expression; inhibits the migration of glioblastoma	Kim *et al*, 2022(52); Xu *et al*, 2022(93)

^a^Concentrations (Conc) are approximate IC_50_ or effective doses from cited studies. Phase color coding is as follows:

^b^blue, G0/G1 dominant;

^c^green, G2/M dominant;

^d^amber, limited/partial efficacy. HCC, hepatocellular carcinoma; TNBC, triple-negative breast cancer; NSCLC, non-small cell lung cancer; GBM, glioblastoma multiforme; BBB, blood-brain barrier.

## Data Availability

Not applicable.
